# Home blood pressure monitoring detects unrevealed hypertension in women with a history of preeclampsia: Results of the BP-PRESELF study

**DOI:** 10.1016/j.ajpc.2022.100429

**Published:** 2022-11-11

**Authors:** Hella E.C. Muijsers, Pensee Wu, Olivier W.H. van der Heijden, Lia D.E. Wijnberger, Chantal van Bijsterveldt, Ciska Buijs, Jens Pagels, Peter Tönnies, Susanne Heiden, Nel Roeleveld, Angela H.E.M. Maas

**Affiliations:** a Radboud University Medical Center, Department of Cardiology, Geert-Grooteplein Zuid 10, 6525 GA Nijmegen, the Netherlands; b Keele Cardiovascular Research Group, School of Medicine, Keele University, Staffordshire ST5 5BG, UK; c Academic Unit of Obstetrics and Gynecology, University Hospital of North Midlands, Stoke-on-Trent ST5 6QG, UK; d Radboud University Medical Center, Department of Obstetrics and Gynecology, Geert-Grooteplein Zuid 10, 6525 GA Nijmegen, the Netherlands; e Rijnstate, Department of Obstetrics and Gynecology, Wagnerlaan 55, 6815 AD Arnhem, the Netherlands; f Canisius-Wilhelmina Hospital, Department of Obstetrics and Gynecology, Weg door Jonkerbos 100, 6532 SZ Nijmegen, the Netherlands; g Maasziekenhuis Pantein, Department of Obstetrics and Gynecology, Dokter Kopstraat 1, 5835 BV Beugen, the Netherlands; h St. Josef Hospital Moers, Department of Obstetrics and Gynecology, Asberger Strasse 4, 47441 Moers, Germany; i Bethanien Hospital Moers, Department of Obstetrics and Gynecology, Bethanienstrasse 21, 47441 Moers, Germany; j St. Antonius Hospital Kleve, Department of Obstetrics and Gynecology, Albersallee 5-7, 47533 Kleve, Germany; k Radboud University Medical Center, Radboud Institute for Health Sciences, Department for Health Evidence, Geert-Grooteplein Zuid 10, 6525 GA Nijmegen, the Netherlands

**Keywords:** Home blood pressure monitoring, Hypertensive disorders of pregnancy, Hypertension, Prevention

## Abstract

**Objectives:**

The risk of cardiovascular disease more than doubles after hypertensive disorders of pregnancy. As early onset chronic hypertension contributes to cardiovascular risk, implementation of screening strategies, using home blood pressure monitoring (HBPM), may help to improve long-term cardiovascular health.

We evaluated whether HBPM among women with a history of preeclampsia/HELLP syndrome is feasible for early detection and management of hypertension.

**Methods:**

The BP-PRESELF study is a multicenter randomized controlled trial. Participants were randomized to intervention group with HBPM for the duration of 1 year or the control group with 'usual care'. The primary outcome was feasibility of HBPM during 1 year of follow-up, defined as protocol adherence, protocol persistence and patient acceptance. Secondary outcomes were blood pressure levels and prevalence of hypertension.

**Results:**

We recruited 198 women with a mean age of 45 years. Protocol adherence decreased during the first 6 months, after which it stabilized. Protocol persistence remained high throughout follow-up. During the study period, 33 women (34%) in the intervention group were diagnosed with hypertension versus only 10 women (11%) in the control group, *P*<0.001. At 1-year follow-up, mean systolic blood pressure (SD) was 120.4 (11.6) mmHg in the intervention group versus 126.1 (14.3) mmHg in the control group, *P*=0.003. Mean diastolic blood pressure (SD) values were 77.1 (8.0) mmHg versus 81.7 (9.4) mmHg, *P*<0.001, respectively. Adjusted systolic and diastolic differences (95% confidence interval) were -6.81 (-10.17, -3.45) and -4.93 (-7.26, -2.61) mm Hg, with 80% less hypertension at 1-year follow-up in the intervention group.

**Conclusions:**

HBPM appears to be feasible for follow-up of blood pressure in women after preeclampsia/HELLP syndrome, while it detected hypertension and blood pressure levels reduced in one-third of women in this group.

## Highlights

• Long-term follow-up of cardiovascular risk in women after preeclampsia is lacking.
• Home blood pressure monitoring seems feasible for early detection of hypertension.
• Home blood pressure monitoring reduced blood pressure values.
• The occurrence of undiagnosed hypertension is reduced.
• Home blood pressure monitoring facilitates long-term follow-up in women after preeclampsia.

## Graphical abstract

**Figure:**
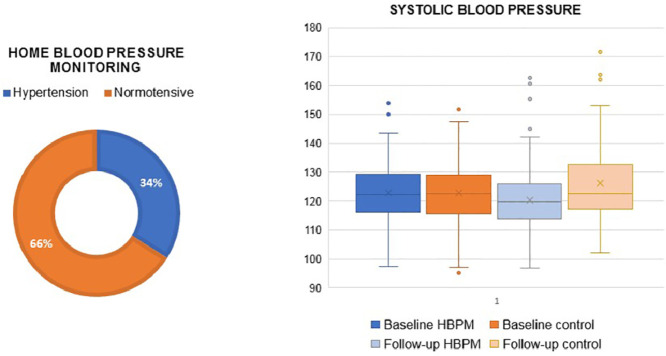

## 1 Introduction

Hypertensive disorders of pregnancy (HDP), such as preeclampsia and HELLP syndrome are severe pregnancy complications, with an over twofold elevated risk of developing cardiovascular disease (CVD) later in life [1], [2], [3]. In women with a prior HDP, several traditional cardiovascular risk factors are associated with the excess CVD risk [4]. Modifiable risk factors, such as elevated blood pressure (BP) and body mass index (BMI), appear to play a role in both the development of HDP and the elevated cardiovascular risk afterwards [5,6]. Early-onset chronic hypertension after HDP is an important contributor to cardiovascular risk [7,8], and anticipate that timely lowering of blood pressure can significantly reduce this risk [9,10].

Several international prevention guidelines recommend BP screening in women after HDP, especially after preeclampsia/HELLP syndrome, although the how and when remains undetermined [[11], [12]]. The use of home blood pressure monitoring (HBPM) may be a good option to overcome this challenge [13]. In patients with hypertension, HBPM was shown to be effective in lowering BP and improving adherence to antihypertensive medication use [14], [15], [16]. In the recent joint American Heart Association/American Medical Association statement, HBPM is advised to confirm the diagnosis of resistant hypertension, to establish white-coat or masked hypertension, and to evaluate response to antihypertensive medication [[17], [18]]. In clinical practice, HBPM is rarely used for the initial diagnosis of hypertension, although its diagnostic ability was already proven a decade ago [19]. HBPM is widely available, easy-to-use, and supports long-term follow-up of BP values. These advantages of HBPM over ambulatory blood pressure monitoring (ABPM) or in-office BP measurements make HBPM more attractive for monitoring those at increased risk of developing hypertension at a young age, such as women affected by HDP.

In this study, we aimed to evaluate whether HBPM in women with a history of preeclampsia/HELLP syndrome is feasible for early detection and management of hypertension.

## 2 Materials and methods

### 2.1 Design and study population

The Blood Pressure after PREeclampsia by SELF monitoring (BP-PRESELF) study is a multicenter randomized controlled trial, conducted between 2017-2019 at the Cardiology department of the Radboud University Medical Center, Nijmegen, the Netherlands. A detailed description of the study protocol was published previously [20].

The BP-PRESELF study was designed to evaluate the feasibility and usability of HBPM to detect hypertension in women with a prior history of preeclampsia/HELLP syndrome.

In brief, women aged 40-60 years with a history of preeclampsia and/or HELLP syndrome more than 1 year ago were recruited from seven Obstetric departments in the European region Rijn-Waal, in the Netherlands and Germany. Previous preeclampsia was defined as diastolic BP (DBP) ≥90 mmHg and/or systolic BP (SBP) ≥140 mmHg and proteinuria ≥300 mg/24h according to the ISSHP definition at that time [21]. Women with a history of eclampsia were also eligible for participation in the current study. Being already diagnosed with hypertension, using antihypertensive medication or previous cardiovascular events were exclusion criteria.

The study was approved by the Regional Committee on Research Involving Human Subjects Arnhem-Nijmegen (number 2016-3006). This trial was registered in the Clinical Trials Register, NCT 03228082, https://clinicaltrials.gov/ct2/show/NCT03228082. The trial was conducted in accordance to the Declaration of Helsinki [22]. All participants gave written informed consent before inclusion.

### 2.2 Measurements

At baseline visit, questionnaires on medical and obstetric history were completed, as well as questionnaires on social functioning and work ability. Furthermore, a physical examination consisting of measurements of BP at least twice at both arms in sitting position, heart rate, weight, height, and waist-/hip circumference was conducted. At this visit, a non-fasting blood sample was also taken and frozen at minus 80⁰C for future biomarker analysis. We were not able to determine glucose and lipid levels due to financial restrictions and focused on feasibility of HBPM as the primary endpoint.

The participating women were randomized to either HBPM, the intervention group, or the control group receiving ‘usual care’. Women in the intervention group received a home BP monitor (Withings) and were asked to perform measurements of their BP for 7 consecutive days each month over one year, according to the recommendations of the European Society of Cardiology/European Society of Hypertension (ESC/ESH) guidelines [23]. Blood pressure measurements were preferably done twice a day, in the morning and evening. The results were automatically uploaded into an online patient health file (Patients Know Best®), accessible to the study coordinators. The weekly average blood pressure was calculated and used to evaluate the presence of hypertension. The women in the intervention group received monthly feedback on their BP measurements, and were given lifestyle advice, if necessary. In case of elevated BP, participants were referred to their GP. Hypertension was defined as SBP ≥135 mmHg and/or DBP ≥85 mmHg for measurements at home, and as SBP ≥140 mmHg and/or DBP ≥90 mmHg for in-office measurements [23].

After 6 months and 1 year of follow-up, women in the intervention group completed questionnaires on feasibility and usability of this regimen of HBPM to evaluate acceptance of the intervention. The exploratory feasibility questionnaire was non-validated and consisted of 9 statements. The usability of the intervention was measured using the 10-item validated system usability scale [24]. Both questionnaires were based on a 5-point Likert scale, with answers varying from ‘strongly disagree’ to ‘strongly agree’. Each answer was given a score from 0 through 4, while the scoring for items that were stated in the opposite direction was reversed. Finally, all scores were summed up per questionnaire and converted to a final score between 0 and 100. Scores close to 100 indicated high patient acceptance.

Women in the control group had no planned BP measurements during follow-up, but were asked to register their BP if measured at home or at a doctor's visit. After 1-year follow-up, all women were invited for a final visit, in which all measurements and questionnaires were repeated in both groups.

### 2.3 Outcomes

The primary outcome of our study was the feasibility of HBPM during 1-year of follow-up. Patient acceptance assessed by self-reported feasibility and usability was determined as subjective measure for feasibility, in addition to protocol adherence and protocol persistence. Protocol adherence was defined as the proportion of participants in the intervention group measuring their BP twice daily for at least 4 consecutive days within every month during 1 year follow-up. Protocol persistence was the proportion of participants in the intervention group who measured their BP every month until final follow-up visit.

The secondary outcomes were BP levels and prevalence of cardiovascular risk factors, such as hypertension, smoking, and BMI in the intervention group compared to the control group at 1-year follow-up.

### 2.4 Data analysis

The Stata/MP version 15.0 and IBM SPSS version 25.0 statistical packages were used for all analyses. The continuous variables were evaluated for normality using skewness and kurtosis. Normally distributed data were shown as means and standard deviation (SD), non-normally distributed data as medians with minimum to maximum range (range), and categorical data as absolute values with percentages. Differences between groups were tested using independent samples *t*-tests, Mann-Whitney *U*-tests, or chi-square tests, respectively. Kaplan-Meier plots were used to examine protocol adherence and persistence, while linear and log-binomial regression analyses were performed to estimate beta coefficients (beta) and relative risks (RR) with 95% confidence intervals (95% CI) as effect measures of HBPM, adjusted for potential confounders.

## 3 Results

**Fig. 1:**
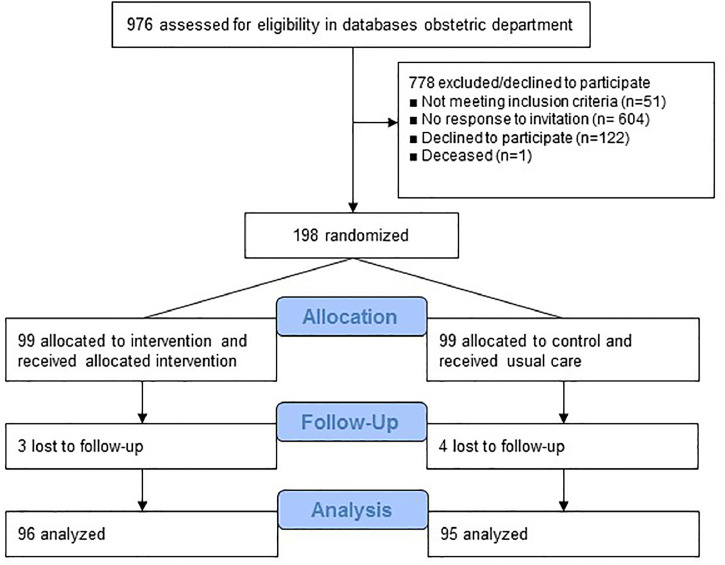
CONSORT flowchart of phases randomized controlled trial.

In total, 198 women with a mean (SD) age of 45 (3.7) years, who were on average 12 (4.3) years after their index pregnancy, were included in the study. Ninety-nine women were randomized to the HBPM group and 99 women to the control group. Of all 198 participants, 7 women did not complete the 1-year follow-up (Fig. 1).

The participants in the intervention group that were lost to follow-up all discontinued intervention before initiation of the first measurement period. Therefore, all results presented are for participants who completed the 1-year follow-up. Baseline characteristics did not show statistically significant differences between groups, except for years since index pregnancy (Table 1). Women in the intervention group were 13 (4.5) years after the index pregnancy, while women in the control group were 11.5 (3.9) years after the index pregnancy (*P*=0.01). However, the women in both groups had similar mean ages at time of inclusion (*P*=0.69). Consequently, women in the intervention group were slightly younger at time of the index pregnancy compared to women in the control group (31.7 versus 32.6 years, respectively, *P*=0.11). No differences were seen in pregnancy-related characteristics, but twice as many women in the intervention group were postmenopausal (*P*=0.14). The mean BP levels at time of inclusion, both systolic and diastolic, were very similar for the intervention group and the control group: 122.7 (11.5) mm Hg versus 122.8 (11.6) mm Hg for SBP (*P*=0.95) and 79.9 (8.1) mm Hg versus 79.5 (8.2) mm Hg for DBP (*P*=0.78), respectively. In the control group, however, 9 women had BP levels consistent with hypertension at the baseline visit. The mean values for heart rate, BMI, and hip-waist ratio were identical in the two groups. In the baseline questionnaire, 4 women (4%) in the intervention group and 10 women (11%) in the control group reported to have been suspected of hypertension at some point in time. Further exploration showed that these diagnoses were made during pregnancy or in the first 3 months afterwards. In the years after the index pregnancy, these diagnoses were not confirmed and BP was no longer treated or controlled. One woman in the intervention group reported the use of beta-blockers for chronic migraine, which was initiated after inclusion but before randomization. Remarkably, over 10% of women in both groups reported chest pain in the last 6 months.

**Table 1:** Demographic and clinical characteristics at baseline.

	Intervention group (n = 96)	Control group (n = 95)	P-value
Age (years)	45.5 (3.9)	45.3 (3.6)	0.69
Caucasian race (%)	92 (96)	93 (98)	0.61
Married (%)	84 (88)	87 (92)	0.56
Maternal age at index pregnancy	31.7 (4.1)	32.6 (3.8)	0.11
Years since index pregnancy	13.0 (4.5)	11.5 (3.9)	0.01
Birth weight index child (grams)	2581.3 (753.7)	2540.3 (972.9)	0.75
Gestational age of index pregnancy (weeks)	37 (22-41)	37 (22-42)	0.93
Number of pregnancies	2 (1-9)	2 (1-6)	0.18
Systolic blood pressure (mm Hg)	122.7 (11.5)	122.8 (11.6)	0.95
Diastolic blood pressure (mm Hg)	79.9 (8.1)	79.5 (8.2)	0.78
Heart rate (bpm)	65.8 (7.8)	65.7 (7.7)	0.95
Body mass index (kg/m²)	25.9 (5.1)	26.0 (4.7)	0.94
Waist-hip ratio	0.80 (0.06)	0.80 (0.07)	0.70
Self-reported previous hypertension (%)	4 (4)	10 (11)	0.09
Antihypertensive medication (%)	1 (1)	0 (0)	0.32
Chest pain in past 6 months (%)	15 (16)	11 (12)	0.42
Diabetes (%)	2 (2)	1 (1)	0.57
Ever diagnosed with venous thromboembolism (%)	2 (2)	1 (1)	0.57
Anticoagulant medication (%)	1 (1)	0 (0)	0.32
Postmenopausal (%)	12 (13)	6 (6)	0.14
Currently smoking (%)	7 (7)	5 (5)	0.56

Data are mean (SD), median (min-max range) or *n* (%). *P*-values are based on independent samples t-tests, Mann-Whitney-U tests, or Chi-square tests, as appropriate.

Table 2 shows the level of patient acceptance of HBPM according to the ESC/ESH recommendation in the intervention group. For both feasibility and usability, the scores were close to 3 out of 4 for most items and between 65 and 74 for the total scores. During the follow-up period, the total feasibility score as well as several sub-scores increased slightly. The participants liked to use the BP monitor, which in general worked well and was easy-to-use. HBPM was especially preferred over in-office measurements in the first 6 months of the study (*P*=0.002). Participants were confident in interpreting the BP values themselves, which further improved towards the end of the study (*P*=0.001). However, the participants indicated that measuring their blood pressure did not result in lifestyle changes, with scores close to 1. The system usability was high throughout follow-up. Participants found it easy-to-learn to use the HBPM and would like to use the system frequently in the future. The scores on complexity of the system shifted during follow-up (*P*=0.05 and *P*=0.03), indicating that the HBPM might have been slightly difficult to use.

**Table 2:** Patient acceptance on feasibility and usability at 6 months and 12 months.

	6 months Mean (SD)	12 months Mean (SD)	P-value
Feasibility score			
I like to use the blood pressure monitor at home to check my blood pressure.	2.86 (1.03)	3.00 (0.97)	0.14
I would not prefer to go to the GP/hospital to check my blood pressure.*	3.39 (0.76)	3.09 (1.00)	0.002
The blood pressure monitor was working well.	2.92 (1.09)	2.95 (0.98)	0.77
I was less worried about my blood pressure while using self-measurements.	2.49 (1.13)	2.52 (1.11)	0.72
Measuring for 7 days in a row, was easy to keep up.	2.72 (1.09)	2.72 (1.03)	0.67
It was easy to remember to measure my blood pressure.*	2.57 (1.10)	2.46 (1.03)	0.65
I think I can interpret the measurements by myself, without receiving feedback every month.	2.31 (1.15)	2.89 (0.94)	<0.001
Measuring my blood pressure did not cause unnecessary agitation/stress.*	3.09 (0.96)	3.05 (1.10)	0.50
The BP results causes lifestyle changes.	1.12 (1.13)	1.22 (1.14)	0.44
Total score	65.19 (13.39)	66.38 (12.35)	0.17
System usability score			
I think that I would like to use this system frequently.	2.30 (1.08)	2.43 (1.08)	0.30
I did not think the system unnecessarily complex.*	3.48 (0.64)	3.36 (0.65)	0.05
I thought the system was easy to use.	3.29 (0.89)	3.18 (0.85)	0.19
I do not think that I would need the support of a technical person to be able to use this system.*	3.30 (0.81)	3.26 (0.80)	0.61
I found the various functions in this system were well integrated.	2.52 (0.72)	2.46 (0.75)	0.23
I did not think there was too much inconsistency in this system.*	2.72 (0.84)	2.72 (0.71)	0.82
I would imagine that most people would learn to use this system very quickly.	3.01 (0.70)	3.00 (0.70)	0.75
I did not find the system very cumbersome to use.*	3.32 (0.67)	3.24 (0.71)	0.27
I felt very confident using the system.	2.41 (0.96)	2.36 (0.96)	0.75
I did not need to learn a lot of things before I could get going with this system.*	3.33 (0.64)	3.18 (0.83)	0.03
Total score	74.22 (11.68)	72.97 (11.23)	0.13

Data are shown as mean ± SD. Each item was scored 0 through 4 with a maximum possible score of 4 for each item. *P*-values are based on paired samples T-test.

⁎ For ease of interpretation of the scores for this statement, the wording from the questionnaire was reversed in the table.

**Fig. 2:**
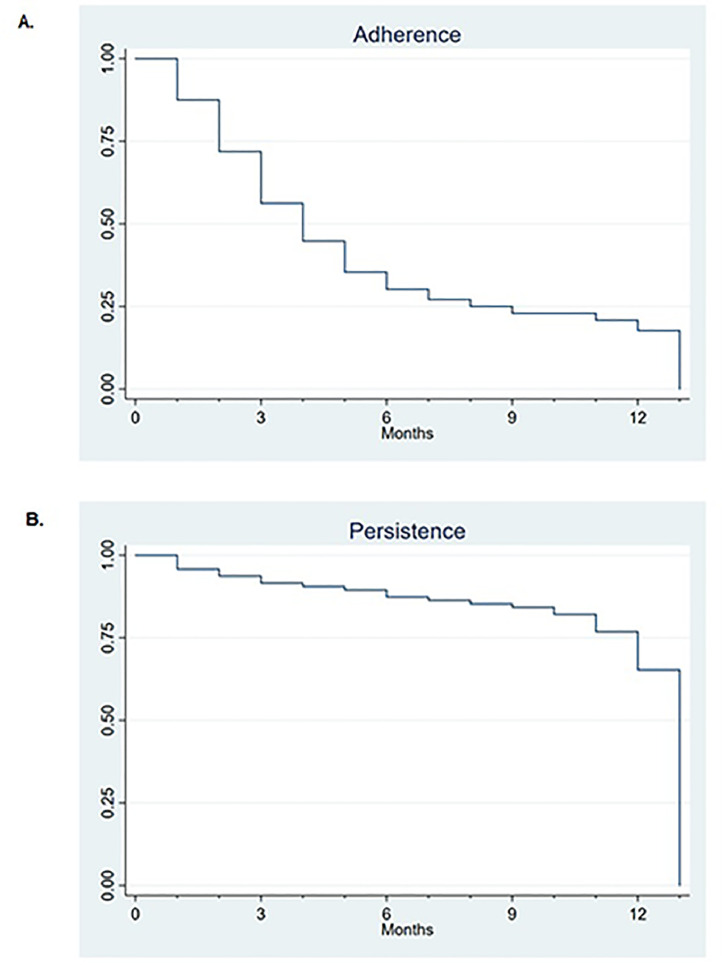
Panel A: survival analysis for protocol adherence. Panel B: survival analysis for protocol persistence.

Fig. 2 shows that protocol adherence (panel A) decreased in the first 6 months of follow-up, after which it stabilized around 25%. However, protocol persistence remained high (>75%) throughout follow-up (panel B). Taking system failure into account, 95.8% of the participants in the intervention group measured their BP at least monthly until end of follow-up.

Table 3 shows the secondary outcomes at the 1-year follow-up visit. During follow-up, 33 women (34%) in the intervention group were newly diagnosed with hypertension and were given lifestyle advice and/or referred to their GP for further control. In six of these women, antihypertensive medication was initiated during follow-up. The other 27 women received lifestyle advice only, such as weight reduction, daily physical exercise, salt restriction, healthy diet, and smoking cessation. Participants were also referred to the website of the Dutch Heart Foundation to read more information. This was sufficient to regulate BP for 24 women in the intervention group. In the control group, only one woman was newly diagnosed with hypertension between initial visit and follow-up visit, while 9 women in this group had high BP levels at the intake visit. Although they were referred to their GP for follow-up, none of these women were actually treated for hypertension during the study period and still had elevated BP levels at the 1-year follow-up visit. In total, twenty women (21%) in the control group were hypertensive at this visit, compared to only five women (5%) in the intervention group, *P*=0.001. All other participants in the intervention group with elevated BP at some point during the follow-up period were normotensive at the 1-year follow-up after lifestyle advice and/or antihypertensive medication. As a result, both SBP and DBP were lower in the intervention group at 1-year follow-up compared to the control group. SBP was 120.4(11.6) mmHg in the intervention group versus 126.1(14.3) mmHg in the control group, *P*=0.003. The DBP values were 77.2(8.0) mmHg versus 81.7(9.4) mmHg, *P*<0.001, respectively.

**Table 3:** Outcomes at 1-year follow-up.

	Intervention group (n = 96)	Control group (n = 95)	P-value
Age (years)	46.6 (3.9)	46.3 (3.6)	0.64
Systolic blood pressure (mm Hg)	120.4 (11.6)	126.1 (14.3)	0.003
Diastolic blood pressure (mm Hg)	77.1 (8.0)	81.7 (9.4)	<0.001
Heart rate (bpm)	64.6 (7.1)	66.7 (8.4)	0.06
Body mass index (kg/m²)	26.0 (5.2)	26.1 (4.9)	0.97
Waist-hip ratio	0.80 (0.06)	0.81 (0.07)	0.58
Hypertension at any time during the study period (%)	33 (34)	10 (11)	<0.001
Hypertension at end of follow-up (%)	5 (5)	20 (21)	0.001
Antihypertensive medication (%)	7 (7)	2 (2)	0.09
Chest pain in last 6 months (%)	9 (9)	14 (15)	0.25
Diabetes (%)	2 (2)	1 (1)	0.57
Ever diagnosed with venous thromboembolism (%)	1 (1)	2 (2)	0.55
Anticoagulant medication (%)	0 (0)	0 (0)	NA
Postmenopausal (%)	17 (18)	8 (8)	0.06
Currently smoking (%)	9 (9)	6 (6)	0.43

Data are mean (SD) or *n* (%). *P*-values are based on independent samples t-tests or Chi-square tests, as appropriate.

In Table 4, the effect estimates for the effects of the intervention on the main secondary outcomes are presented, adjusted for confounding by years since index pregnancy, menopausal status, and BMI at 1-year follow-up. These results underscore the positive effects of HBPM on both SBP and DBP with differences of -6.81 (-10.17, -3.45) and -4.93 (-7.26, -2.61) mmHg, respectively compared to the control group. Women in the intervention group had an almost 3 times higher chance of a diagnosis of hypertension during the study period (RR_adj_ = 2.80; 95% CI: 1.45, 5.42). Consequently, hypertension occurred 80% less often in the intervention group compared to the control group at the 1-year follow-up visit (RR_adj_ = 0.20; 95% CI: 0.08, 0.51). Use of antihypertensive medication was 3 times higher in the intervention group, whereas the number of women with reported chest pain was almost 40% less compared to the control group.

**Table 4:** Effect estimates for the effects of the intervention on the main secondary outcomes at 1-year follow-up.

	Beta coefficient / RR (95% CI)	Adjusted effect estimates
Beta coefficient		
Average systolic blood pressure	-5.71 (-9.44, -1.99)	-6.81 (-10.17, -3.45)
Average diastolic blood pressure	-4.54 (-7.03, -2.05)	-4.93 (-7.26, -2.61)
Body mass index	-0.03 (-1.47, 1.42)	-0.31 (-1.77, 1.15)
Waist-hip ratio	0.00 (-0.01, 0.02)	0.00 (-0.02, 0.02)
Relative risk		
Hypertension at any time during the study period	3.27 (1.71, 6.24)	2.80 (1.45, 5.42)
Hypertension at end of follow-up	0.25 (0.10, 0.63)	0.20 (0.08, 0.51)
Antihypertensive medication	3.46 (0.74, 16.25)	2.95 (0.63, 13.91)
Chest pain	0.64 (0.29, 1.40)	0.62 (0.27, 1.39)

Data are beta coefficients (95% CI) for continuous outcomes and RRs (95% CI) for categorical outcomes comparing the intervention group to the control group. Adjusted effect estimates were adjusted for years since index pregnancy, menopausal status, and BMI (except in analyses for BMI and waist-hip ratio) at 1-year follow-up.

## 4 Discussion

In this intervention study, HBPM was shown to be feasible for long-term follow-up of BP in women at elevated risk of developing hypertension at an early age. However, strict adherence to the protocol was importantly reduced after 6 months, with only 17 women in the intervention group (17.7%) being adherent to the over the entire 1-year follow-up, measuring for at least 4 consecutive days every month. Nevertheless, the majority of women in the intervention group stayed motivated to measure their BP regularly. The use of monthly reminders by email or phone messaging may have been helpful. HBPM detected hypertension and clearly reduced BP levels in the intervention group compared to the control group.

To the best of our knowledge, this is the first study evaluating HBPM in women at intermediate term after their diagnosis of HDP. Studies evaluating HBPM during hypertensive pregnancies have shown that self-monitoring leads to fewer antenatal visits with comparable fetal, neonatal, and maternal outcomes [25]. However, it is still debated when and how cardiovascular screening after HDP should be conducted. Several studies recommend the initiation of screening during the first decade after HDP [26], whereas others recommend screening immediately following the complicated pregnancy [[27], [28]]. It also remains to be elucidated whether HBPM or intermittent 24-hours ABPM or even a combination of the two are the most optimal ways for follow-up [[29], [30]]. An advantage of HBPM may be that women who are already trained to use HBPM during pregnancy, can easily be instructed to use BP monitoring at home in the years afterwards. There is a lack of evidence on trajectories of cardiovascular risk in women with HDP which limits risk stratification [31]. Therefore, long-term follow-up using HBPM may help to reduce this knowledge gap. Lagerweij et al. [32] showed that early cardiovascular risk screening and lifestyle interventions lead to long-term health benefits, albeit with health economic implications. HBPM may be a cost-effective method to identify women at highest risk who benefit from a cardiovascular screening program and lifestyle interventions.

A huge advantage of HBPM nowadays is fewer time-consuming in-person clinic visits, with more involvement of the patients themselves to control their health risk. The recent COVID-19 pandemic has demonstrated that self-management and remote consultations with clinicians are valuable and easy-to-implement tools in healthcare.

We observed that the system usability score for complexity of the system decreased statistically significantly during follow-up, but the scores remained high overall. During follow-up, thirteen women experienced problems with the BP monitor, mostly caused by failing Bluetooth connections to the mobile phone. These participants received new BP monitors, which resolved their issues. This might explain the lower scores on complexity and need for support in the second half of the follow-up period. Despite the slightly lower scores, overall scores on feasibility and usability of the intervention were still high at the end of follow-up, indicating patient satisfaction with HBPM. The score on interpretation increased, which suggest participants went through a positive learning curve. Most women indicated that they felt confident in interpreting BP values at the 1-year follow-up visit, especially since elevated BP values were color-coded in their mobile application.

Our study confirms that there is a high prevalence of yet unknown hypertension in young middle-aged women after HDP, which approximated to 30% of study participants at any point during follow-up. As we excluded women who were already known with hypertension, the actual number of hypertensive women is even higher. In the baseline questionnaire, 14 women (7%) reported to have had hypertension since the index pregnancy, although physical examination showed these women to be normotensive at enrollment. The women were informed that they had hypertension during their pregnancy or within the 3-months postpartum period, but they did not have any treatment for BP in the intervening years.

Although mean age at inclusion was comparable in the two study groups, twice as many women were postmenopausal in the intervention group. While this most likely will not affect feasibility of HBPM, it might have biased the comparison of BP levels between the two groups, since BP rises in (peri)menopausal women and the risk of developing high BP increases [33]. By adjusting the final results for menopausal status, this bias was removed, unless residual confounding still led to slight underestimation of the effects of HBPM on BP levels.

The main strengths are the randomized controlled design and the relatively large sample size. One of the limitations is that we were not able to confirm the diagnosis of hypertension using ABPM. However, several studies already confirmed the high diagnostic agreement between these two methods [19]. Furthermore, the results of the BP-CHECK study will provide new knowledge on accuracy of several different BP measuring methods [34]. We cannot rule out that a small part of the between group difference in prevalence of hypertension at end of follow-up is the result of regression to the mean. However, evidence shows that regression to the mean occurs in both HBPM and office BP measurements [35]. We cannot rule out that our results were affected by selection, especially in participant recruitment. Of those excluded from the study, most did not respond to the study invitation and were not reachable via mail or phone. Moving houses and jobs during the prolonged period after the index pregnancy was the major reason that we were not able to contact these patients. Therefore, we assume that our results can be extrapolated to the target population without much error.

## 5 Conclusions

Our findings confirm that HBPM is feasible for follow-up of BP in women at increased risk of developing hypertension early in life. Further research is needed to determine the optimal way to measure BP at home to detect hypertension in a timely fashion in women after their diagnoses of HDP.

## Authors’ contributions

HM, OH, NR, AM contributed to the conception or design of the work. HM, PW, NR contributed to data analysis and interpretation. HM and AM drafted the manuscript. HM, PW, OH, LW, CvB, CB, JP, PT, SH, NR and AM critically revised the manuscript. All gave final approval and agree to be accountable for all aspects of work ensuring integrity and accuracy.

## Declaration of Competing Interest

The authors declare that they have no known competing financial interests or personal relationships that could have appeared to influence the work reported in this paper.
